# Dynamic social power modulates neural basis of math calculation

**DOI:** 10.3389/fnhum.2012.00350

**Published:** 2013-02-06

**Authors:** Tokiko Harada, Donna J. Bridge, Joan Y. Chiao

**Affiliations:** ^1^Department of Psychology, Northwestern UniversityEvanston, IL, USA; ^2^Department of Psychology, Interdepartmental Neuroscience Program, Northwestern UniversityEvanston, IL, USA

**Keywords:** math achievement, social class, priming, inferior frontal gyrus, fMRI

## Abstract

Both situational (e.g., perceived power) and sustained social factors (e.g., cultural stereotypes) are known to affect how people academically perform, particularly in the domain of mathematics. The ability to compute even simple mathematics, such as addition, relies on distinct neural circuitry within the inferior parietal and inferior frontal lobes, brain regions where magnitude representation and addition are performed. Despite prior behavioral evidence of social influence on academic performance, little is known about whether or not temporarily heightening a person's sense of power may influence the neural bases of math calculation. Here we primed female participants with either high or low power (LP) and then measured neural response while they performed exact and approximate math problems. We found that priming power affected math performance; specifically, females primed with high power (HP) performed better on approximate math calculation compared to females primed with LP. Furthermore, neural response within the left inferior frontal gyrus (IFG), a region previously associated with cognitive interference, was reduced for females in the HP compared to LP group. Taken together, these results indicate that even temporarily heightening a person's sense of social power can increase their math performance, possibly by reducing cognitive interference during math performance.

## Introduction

### Power and cognition

Priming social power has been shown to affect both social and cognitive processing. People with low power (LP) typically experience heightened uncertainty and increased vigilance of the social environment (for review, see Keltner et al., 2003). Prior behavioral studies have shown that priming people with LP increases their sensitivity to other people's perspectives—likely due to the tendency to be concerned with performance evaluations given by their superiors (Galinsky et al., 2006). As a consequence of heightened social vigilance, neural resources typically recruited to carry out a set of cognitive functions may be taxed, leading to suboptimal performance on cognitive tasks.

One cognitive mechanism affected by situational power is local-global attentional processing. Because LP individuals attempt to attend to an overabundance of information in the environment, their perception of the big picture or global meaning may be hindered as a result of their attentional focus on many small, local details. When participants' situational power was modulated while completing an attentional scope during a hierarchical attention task, (i.e., Navon figures; Navon, 1977), LP participants demonstrated a local processing preference, such that their reaction time to detect local cues was significantly faster than for global cues. By contrast, high power (HP) participants identified the local and global targets with equal speed.

LP participants' global focus may be impeded by a heightened susceptibility to interference of the local components. Their inability to filter out extraneous information efficiently may be a reflection of depleted executive functioning resources. This idea is supported by behavioral evidence that showed a relatively exaggerated interference effect on a Stroop task and an N-back task, with LP participants making more errors than HP participants on both tasks (Smith et al., 2008). Relatedly, when members of stigmatized groups are reminded of their low status, they show impaired working memory (Spencer et al., 1999; Schmader and Johns, 2003; Beilock et al., 2007). Specifically, when reminded of negative gender stereotypes about math (e.g., women are bad at math), they are more susceptible to reduced working memory capacity and subsequent worse performance on math tests compared to women who are not reminded of such negative stereotypes (Schmader and Johns, 2003).

### Neural basis of math calculation

Mathematical calculation relies on several distinct cognitive and neural mechanisms underlying numerical processing (Dehaene, 1992; Dehaene et al., 2003). Here we focus on two types of numerical calculations that are subserved by dissociable neural networks and cognitive processes (Dehaene et al., 1999; Stanescu-Cosson et al., 2000) that form the basis for later mathematical achievement in educational settings (Halberda et al., 2008): exact and approximate math calculation. Exact calculation requires explicit rote memory retrieval of solutions that have previously been learned, such as computing the answer to small addition or multiplication problems (e.g., 3 + 4 = 5 or 7). Because the solution is a concrete answer stored in memory, learning is item-specific, such that extensive training on a subset of addition problems shortens response time to these specific problems, but this reaction time benefit does not extend to new, untrained problems (Dehaene et al., 1999). By contrast, approximate calculation does not require retrieval of previously learned material, but instead relies on the comparison of a quantity that fall along a mental number line and which ultimately leads to surprisingly precise estimation judgments. Unlike exact calculations, approximation is a generalized learning process, such that training on a subset of approximate problems leads to faster response times on both trained and untrained problems (Dehaene et al., 1999).

Previous research has established a robust number size effect, with increasing size corresponding to lengthier response times and heightened error rate on basic addition and multiplication problems (for review, see Ashcraft, 1992). Interestingly, the type of math operation, exact or approximate, interacts with problem size, with a notably larger effect of size evident on exact calculations relative to approximate problems (Stanescu-Cosson et al., 2000). Small exact answers may be accessed automatically due to their pronounced salience and associative properties in memory (LeFevre et al., 1988). Small approximate solutions, on the other hand, may take relatively longer to compute because the exact answer produces an interference effect, thus requiring the active inhibition of the exact answer before comparing the relevant answer choices. Notably, neural regions within the parietal lobe have also been shown not only represent numerical distance, but also social status distance. When comparing large and small distances across numerical and status domains, people show increase parietal response for large compared to small distance comparisons, an effect paralleled in response time during numerical and status comparison (Chiao et al., 2009a). These findings indicate that neural representations within the inferior parietal lobe subserve numerous kinds of cognitive and social domains (Chiao, 2010), likely as a function of spatial distance (Cohen Kadosh and Walsh, 2008, 2009).

Here we aimed to investigate the influence of power priming on the neural basis of math calculation. Intact executive functioning may be crucial for some types of mathematical processing. Inefficient executive functioning may impede performance on some types of math calculations, particularly those that require the use of cognitive control mechanisms such as updating, information filtering, and competitive selection processes. For instance, math approximation has been shown to recruit the subregions of the superior parietal lobe, including the intraparietal sulcus (IPS), a region that is also important in magnitude comparisons, such as size and numerosity (Cohen-Kadosh et al., 2008), and even abstract hierarchical social relations, such as social status (Chiao et al., 2009a; Chiao, 2010). On the other hand, exact calculation recruits a network including the inferior frontal gyrus (IFG), a region implicated in attentional control processes such as inhibition, selection and is particularly important when processing verbal material (Aron et al., 2004). Given that differential neural substrates are recruited during the processing of approximate and exact mathematical problems, we hypothesize that priming individuals with either low or HP differentially recruit neural substrates of numerical processing within bilateral IFG and IPS during exact or approximate calculation, respectively.

Since executive function resources are needed to actively inhibit interfering information, we predicted that priming participants with LP would affect performance on approximation problems. Specifically, we hypothesized that LP participants would demonstrate decreased computational efficiency when solving approximate problems relative to HP participants, because they may be more susceptible to cognitive interference when generating an exact answer and thus, require additional recruitment of cognitive control brain regions to exercise inhibition. On the other hand, we did not expect group performance differences on the exact calculations, since the solutions to these problems are likely automatically retrieved from memory.

## Materials and methods

### Participants

Twenty-four right-handed, Caucasian females (*Age in years*: *M* = 20.38, *SE* = 0.33) participated in this study for cash payment. Inclusion criteria included only female participants due to prior demonstration that females demonstrate heightened stigma or stereotype threat during math calculation and thus may demonstrate malleability in math performance as a function of power priming (Spencer et al., 1999; Schmader and Johns, 2003). All participants had normal or corrected-to-normal vision and gave informed consent before completing the study. Half of the participants were randomly assigned to the HP priming group and the other half were assigned to the lower power (LP) priming group. Note: due to behavioral data loss, behavioral analyses were conducted on only 22 participants, half in the HP and half in the LP group.

### Procedures

Before participants arrived to the study site, they were randomly assigned to the HP or LP condition. After completing the appropriate fMRI prescreening paperwork, participants were given instructions and completed the two priming tasks and the math task in the experiment.

#### Power primes

**Figure 1:**
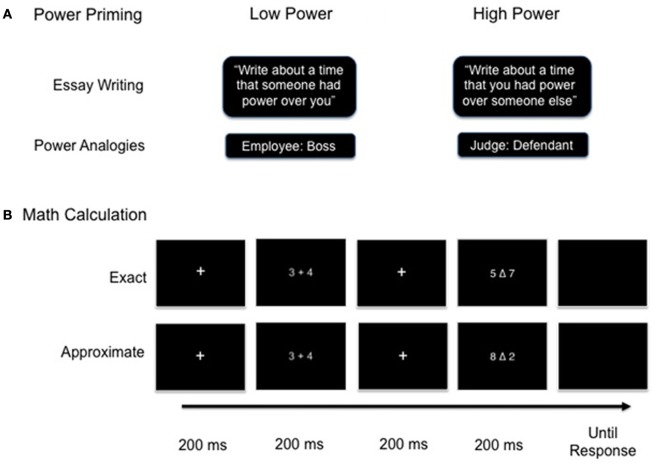
**(A,B)** Experimental design consisting of power priming procedure followed by exact and approximate math calculation task.

Power was primed using two separate procedures. Participants were first primed with an essay-writing task (adapted from Galinsky et al., 2003) in the outside of the scanner. They were asked to complete this task as a “warm-up” before completing the tasks inside the scanner. In this task, participants were asked to reflect upon a personal situation in which they maintained a position of power or powerlessness and write about it for 5 min (Figure 1). After 5 min, the experimenter collected the participant's essay and the participant was taken to the scanner.

Inside the scanner, but prior to scanning, participants completed a second power prime consisting of a power analogy task corresponded to their pre-assigned prime condition (Bridge and Chiao, submitted). The power analogy prime consisted of 24 hierarchical social role pairs displayed in an analogical format (e.g., Teacher: Student, see Figure 1 and **Appendix**). Social role pairs were presented at the top of the screen with four multiple choice selections displayed beneath the roles. Participants were asked to imagine that they occupied the role in the first position and then determine which word best described the relationship between the social roles from their assigned perspective. Four multiple selections were available for participants to choose from; one HP, one LP, and two neutral options. The role in the first position corresponded to the condition assigned to the participant. In the HP condition, the powerful social role was always situated in the first position. Participants took a 1st person perspective and chose the answer that best described how they would see themselves in relation to a person who occupied the role in the second position (e.g., knowledgeable). In the LP condition, the LP social role was located in the first position. Participants took a 3rd person perspective and chose the answer that best described how a person occupying the role in the second position would see them (e.g., impressionable). Participants made a button press to select the most appropriate answer and were unable to move on to the next screen until they chose the correct answer. After making the correct selection, a screen appeared that reinforced their answer choice. Hence, participant's performance on the power priming task was 100% accurate.

#### Math task

A total of 20 small addition problems were used in the math task. Ten small addition problems and corresponding approximate (e.g., 6 + 2 = 3 or 9) and exact (e.g., 6 + 2 = 8) answer choices were administered (adapted from Stanescu-Cosson et al., 2000). An additional ten small problems and answer choices were constructed using the same constraints specified by Stanescu-Cosson et al. (2000) with four problems including ties (e.g., 2 + 2 = 4). Each addition problem had two sets of answer choices: one pair of exact answer choices and one pair of approximate answer choices. Therefore, the same addition problems were used in both math task conditions, with the only variation being in the answer selections. The location of the correct answer choice (left or right of central triangle) was counterbalanced across blocks and conditions.

We employed a block design that included 5 approximate math, 5 exact math, and 11 gray square control blocks. Participants completed alternating blocks of the approximate and exact math conditions with interleaving blocks of the control task during the fMRI scan. The order of the math blocks was counterbalanced across participants, but each functional run always began and ended with the control task. Each block was comprised of eight response trials that followed the same presentation format. For the math tasks, each trial began with a 200 ms central fixation cross followed by the presentation of an addition problem for 200 ms. Next, a central fixation cross was again displayed for 200 ms, after which two answer choices appeared on the screen for 200 ms. Once the answer choices disappeared from the screen, participants were prompted to make a button press response with their right index or middle finger to select the answer choice on the left or the right, respectively. Participants were allotted 2200 ms to make a response, but they were instructed to respond as quickly as possible without sacrificing speed for accuracy. The format of the gray square control task was identical to the math tasks. Rather than viewing an addition problem and two answer choices, participants instead saw two brief presentations of a gray square centered on the screen. During the allotted response time, participants were prompted to press a button their index finger as quickly as they could. The control blocks served as both a rest period and a baseline to subtract neural activity related to motor preparation and execution. Prior to scanning, participants were given practice trials of each condition in order to gain familiarity with the tasks and the timing of each stimulus presentation.

### Behavioral surveys

After scanning, participants completed several behavioral surveys to assess possible individual differences that may affect math calculation, specifically math confidence, explicit math attitudes, and personality traits, such as anxiety (e.g., state-trait anxiety).

### fMRI parameters

Functional brain images were acquired at the Center for Advanced Medical Resonance Imaging (CAMRI) facility located in the Northwestern Medical Hospital in Chicago, IL. Scanning occurred on a 3.0 Tesla Siemens Trio MRI scanner equipped with single-shot, whole-body, echo planar image [repetition time (TR) = 2000 ms; echo time (TE) = 25 ms; flip angle = 70°; FOV = 20 cm, 64 × 64 matrix; 34 slices; voxel size = 3.0 × 3.0 × 4.0 mm], sensitive to BOLD contrast. A high-resolution anatomical T1-weighted image was also acquired [TR = 2300 ms; TE = 2.91 ms; flip angle = 9°; FOV = 256 mm; 256 × 256 matrix; 176 slices; voxel size = 1.0 × 1.0 × 1.0 mm] for each subject. All stimuli were presented using Presentation software (Neurobehavioral Systems, Albany, CA) and projected onto a half-transparent viewing screen located behind the head coil. Subjects viewed the projected stimuli through a mirror.

### fMRI analysis

Functional images were analyzed using SPM5 software (Wellcome Department of Imaging Neuroscience, London, UK) implemented in Matlab (Mathworks, Cherborn, MA, USA). First, all volumes were realigned spatially to the first volume and a mean image was created. After a high-resolution image was coregistered onto the mean image, all volumes were normalized to the MNI (Montreal Neurological Institute) space using a transformation matrix obtained from the normalization process of the high-resolution image of each individual subject to the MNI template. The normalized images were then spatially smoothed with an 8 mm Gaussian kernel.

After preprocessing, statistical analysis for each individual subject was conducted using the general linear model (Friston et al., 1999). At the first level, each block of trials was modeled by convolving with a hemodynamic response function. For individual subjects, a linear regressor was applied to filter noise. In order to test hypotheses about regionally specific condition effects, parameter estimates for each condition were computed using the following linear contrasts: Exact > Control, Approximate > Control, Exact > Approximate, Approximate > Exact.

Random-effect analyses were then conducted with individual subject contrast images (Friston et al., 1999). One-sample *t*-tests were performed for each of the four comparisons described above and results were visualized at an uncorrected threshold of *p* < 0.001, extent threshold of 15 voxels. Next, we computed the interactions of power prime and type of math calculation with two-sample *t*-tests performed on HP_Exact > Approximate_ > LP_Exact > Approximate_, LP_Exact > Approximate_ > HP_Exact > Approximate_, LP_Approximate > Exact_ > HP_Approximate > Exact_, HP_Approximate > Exact_ > LP_Approximate > Exact_, HP_Exact > Control_ > LP_Exact > Control_, LP_Exact > Control_ > HP_Exact > Control_, LP_Approximate > Control_ > HP_Approximate > Control_, HP_Approximate > Control_ > LP_Approximate > Control_. Group analyses were visualized at an uncorrected threshold of *p* < 0.005, extent threshold of 15 voxels.

To further investigate predicted group interaction effects within specific regions-of-interest (fROIs): independent ROIs were defined via main effect comparisons of Approximate > Control and Exact > Control contrasts and functional ROIs were defined by the interaction of power prime and type of math calculation. Each ROI was defined as a sphere with a 10 mm diameter was drawn around each peak voxel that arose from the random effects analysis with a *p* < 0.001 threshold and cluster size of 15. Functional regions-of-interest analyses were performed using Marsbar (Brett et al., 2002) software implemented with SPM5. To identify Brodmann areas and brain regions, MNI coordinates were converted to Talairach using a non-linear transformation (http://imaging.mrc-cbu.cam.ac.uk/imaging/MniTalairach). Brodmann areas and brain regions were identified based on the Talairach Atlas (Talairach and Tournoux, 1988). All coordinates are reported in MNI coordinates here.

## Results

### Behavioral results

#### Accuracy

We conducted a 2 (*Power Prime*: High, Low) × 2 (*Math Calculation*: Exact, Approximate) between-subjects ANOVA with accuracy. There were no main effects or interactions with power on RT (all *p*s > 0.05).

#### Reaction time

We conducted a 2 (*Power Prime*: High, Low) × 2 (*Math Calculation*: Exact, Approximate) between-subjects ANOVA with RT and observed a significant effect of math task on RT, *F*_(1, 22)_ = 31.87, *p* < 0.0001, such that exact calculations were correctly solved faster than were approximate calculations (Table 1). There were no main effects or interactions with power on RT (all *p*s > 0.05).

**Table 1:** **Behavioral results (*M* ± *SE*)**.

	**High power (HP)**	**Low power (LP)**
Reaction time		
Exact	479 (32)	470 (32)
Approximate	567 (28)	553 (28)
Accuracy		
Exact	98% (1%)	99% (1%)
Approximate	99% (1%)	96% (1%)
Math confidence	6.83 (0.64)	6.17 (0.64)
Math attitudes	6.67 (0.53)	4.83 (0.87)^*^
State-trait anxiety inventory		
State anxiety	1.68 (0.14)	1.64 (0.09)
Trait anxiety	1.93 (0.09)	1.80 (0.08)

* p ≤ 0.05.

#### Math confidence

There was no main effect of power prime on math confidence (*p* > 0.05; Table 1).

#### Explicit math attitudes

HP prime participants (*M* = 6.67, *SE* = 0.53) showed more positive attitudes about math compared to LP prime participants (*M* = 4.83, *SE* = 0.87), *t*_(22)_ = 1.08, *p* < 0.05, Table 1).

#### State-trait anxiety

There was no main effect of power prime on state or trait anxiety (*p*s > 0.05; Table 1).

### fMRI results

#### Main effect of math calculation

**Figure 2:**
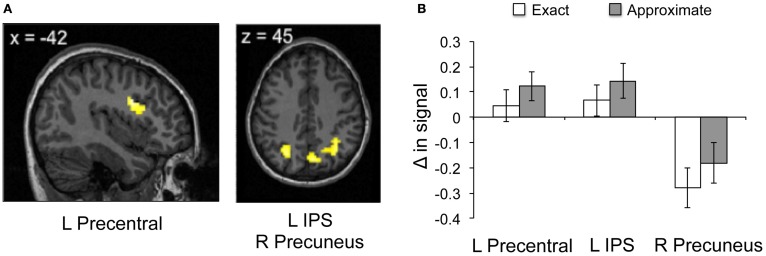
**Neural results during math calculation. (A,B)** Greater neural response within left precentral, left inferior parietal sulcus and right precuneus to approximate compared to exact math calculation.

For all participants, several subregions within the frontal and parietal lobes showed greater neural response during exact math calculation compared to baseline, including the left angular gyrus and bilateral IFG (Figure 2, Table 2). Compared to baseline, exact math calculation revealed greater neural response within left angular gyrus, right superior parietal lobe, right caudate, bilateral IFG, and left anterior cingulate cortex (Figure 2). More specifically, compared to baseline, approximate math calculation revealed greater neural response within bilateral intraparietal sulci (IPS) and bilateral IFG (Table 2), regions previously implicated in exact math processing (e.g., Stanescu-Cosson et al., 2000). Compared to exact math calculation, greater neural response was observed within predicted regions of interest within the frontal and parietal lobes, specifically right precuneus, left IPS and left IFG during approximate math calculation (Figure 2; Table 2). No additional regions showed increased neural response in the reverse contrast of exact compared to approximate math calculation.

**Table 2:** **Main effect of math calculation**.

**Region**	**BA**	**Voxels**	***x***	***y***	***z***	***Z* score**
**EXACT > CONTROL**						
L Angular gyrus	39	474	−27	−62	39	5.96
L Cerebellum		251	−3	−80	−19	5.51
**L Inferior frontal gyrus**	**47**	**327**	**−30**	**26**	**−4**	**5.17**
R Caudate		118	21	−5	20	4.89
**R Superior parietal lobe**	**7**	**129**	**24**	**−62**	**50**	**4.76**
R Cerebellum		78	30	−68	−19	4.75
**R Inferior frontal gyrus**	**47**	**116**	**33**	**29**	**−4**	**4.62**
R Inferior occipital gyrus	19	86	42	−73	1	4.51
L Middle frontal gyrus	6	84	−27	0	50	4.28
L Thalamus		49	−15	−11	17	3.87
R Inferior frontal gyrus	9	26	39	10	24	3.65
L Anterior cingulate cortex	32	16	−6	11	46	3.64
**APPROXIMATE > CONTROL**						
**L Intraparietal sulcus**	**7**	**621**	**−27**	**−59**	**53**	**6.15**
R Cerebellum		509	6	−77	−16	6.07
**R Intraparietal sulcus**	**7**	**289**	**30**	**−56**	**50**	**5.47**
L Fusiform gyrus	37	134	−42	−56	−10	4.95
**L Inferior frontal gyrus**	**47**	**489**	**−30**	**26**	**−4**	**4.81**
R Cingulate cortex	24	369	6	4	27	4.73
L Middle frontal gyrus	6	151	−24	−3	53	4.63
R Precentral sulcus	9	49	39	7	30	4.23
R Middle occipital gyrus	18	26	33	−84	4	4.18
**R Inferior frontal gyrus**	**45/46**	**20**	**33**	**27**	**18**	**4.12**
**R Inferior frontal gyrus**	**47**	**92**	**33**	**23**	**−1**	**4.10**
R Inferior occipital gyrus	19	42	45	−70	1	3.84
**APPROXIMATE > EXACT**						
R Precuneus	7	233	6	−68	48	4.42
**L Inferior frontal gyrus**	**44**	**46**	**−42**	**4**	**27**	**4.19**
R Cerebellum		19	9	−77	−24	3.92
**L Intraparietal sulcus**	**7**	**78**	**−27**	**−62**	**47**	**3.92**
**EXACT > APPROXIMATE**						
No suprathreshold clusters						

p < 0.001 uncorrected; 15 contiguous voxels; MNI coordinates.

#### Main effect of power prime

There was no main effect of power prime on neural response. However, compared to HP participants, LP participants showed greater right precentral gyrus during exact and approximate calculation relative to baseline (Table 3).

**Table 3:** **Main effect of power prime**.

**Region**	**BA**	**Voxels**	***x***	***y***	***z***	***Z* score**
HP_(Approximate + Exact)_ > LP_(Approximate + Exact)_						
No suprathreshold clusters						
LP_(Approximate + Exact)_ > HP_(Approximate + Exact)_						
No suprathreshold clusters						
HP_(Approximate + Exact > Control)_ > LP_(Approximate + Exact > Control)_						
No suprathreshold clusters						
LP_(Approximate + Exact > Control)_ > HP_(Approximate + Exact > Control)_						
Precentral gyrus	6	16	42	−6	27	4.06

p < 0.001 uncorrected; 15 contiguous voxels; MNI coordinates.

#### Interaction of power prime and type of math calculation

HP participants showed greater neural response within right precuneus and left cerebellum compared to LP participants during exact calculation compared to control (Table 4). Compared to HP participants, LP participants showed greater neural response within three regions during approximate calculation compared to control, specifically left anterior insula extending into the IFG, right claustrum, and right precentral gyrus (Table 4). Finally, consistent with our predictions, neural response within the left IFG and right caudate was heightened for LP participants during approximate math calculations and for HP participants during exact math calculations (Table 4). No additional contrasts of interest revealed significant clusters of activation.

**Table 4:** **Interaction of power prime and math calculation**.

**Region**	**BA**	**Voxels**	***x***	***y***	***z***	***Z* score**
HP_(Exact > Control)_ > LP_(Exact > Control)_						
R Precuneus/PCC	31	65	6	−60	22	3.95
L Cerebellum		23	−27	−62	−12	2.89
LP_(Exact > Control)_ > HP_(Exact > Control)_						
No suprathreshold clusters						
HP_(Approximate > Control)_ > LP_(Approximate > Control)_						
No suprathreshold clusters						
LP_(Approximate > Control)_ > HP_(Approximate > Control)_						
L Inferior frontal gyrus	47	18	−33	26	−4	3.44
R Claustrum		123	30	−19	20	3.37
R Precentral gyrus	4	30	33	−15	45	3.25
HP_(Approximate > Exact)_ > LP_(Approximate > Exact)_ or LP_(Exact > Approximate)_ > HP_(Exact > Approximate)_						
No suprathreshold clusters						
LP_(Approximate > Exact)_ > HP_(Approximate > Exact)_ or HP_(Exact > Approximate)_ > LP_(Exact > Approximate)_						
R Caudate nucleus		26	12	−2	22	3.46
**L Inferior frontal gyrus**	**47**	**15**	**−30**	**20**	**−6**	**3.27**

p < 0.005 uncorrected; 15 contiguous voxels; MNI coordinates.

#### ROI analysis

***ROI analysis-Functional.*** To further examine the interaction of power prime and type of math calculation, we examined the neural response within the functionally-defined left IFG ROI, when controlling for individual differences in math confidence, math attitudes and anxiety.

**Figure 3:**
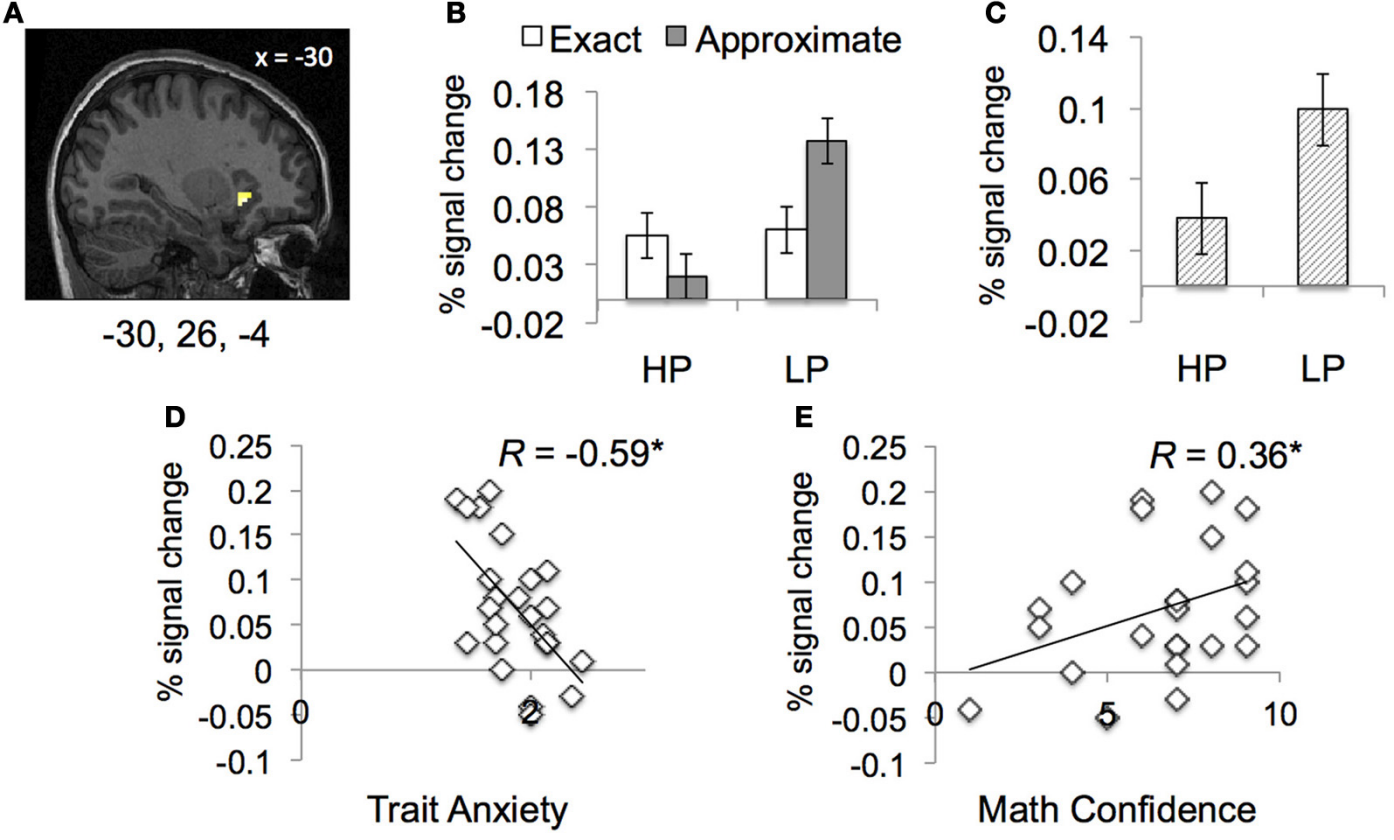
**Neural results in functionally-defined ROI as a function of power prime and type of math calculation. (A,B)** Compared to high power (HP) prime, people with low power (LP) prime show greater response with left IFG during math calculation, particularly when solving approximate math problems. **(C)** Across type of math calculation, people in LP prime group showed greater response in left IFG compared to people in HP prime group. **(D)** People with greater trait anxiety show reduced neural response within left IFG during math calculation. **(E)** People with greater math confidence show increased neural response within left IFG during math calculation. Regression results indicate power priming, trait anxiety and math confidence are unique predictors of neural response within IFG, *R*^2^ = 0.62, *F*_(5, 23)_ = 5.78, *p* < 0.05.

In the left IFG [−30 26 −4], we observed an interaction of power prime and type of math calculation, *F*_(1, 18)_ = 6.55, *p* < 0.05 (Figure 3). Within left IFG, LP participants showed significantly greater neural response during approximate compared to exact math calculation, *t*_(11)_ = 2.81, *p* < 0.02, whereas HP participants showed no difference in neural response within the same region as a function of math calculation. Additionally, there was also a main effect power prime, *F*_(1, 18)_ = 5.19, *p* < 0.05; irrespective of type of math calculation, LP participants showed significantly increased neural response with left IFG compared to HP power participants.

Finally, there was also a significant effect of individual differences in math confidence, *F*_(1, 18)_ = 5.65, *p* < 0.05 and trait anxiety *F*_(1, 18)_ = 4.58, *p* < 0.05 on neural response within left IFG. Across all participants, people who reported greater math confidence, showed greater neural response within left IFG during math calculation, *r*_(24)_ = 0.36, *p* < 0.05. By contrast, across all participants, people who reported greater trait anxiety displayed reduced neural response within left IFG during math calculation, *r*_(24)_ = −0.59, *p* < 0.001.

***ROI analysis-Independent.*** To further examine our hypothesis, we examined neural response within bilateral IFG and bilateral IPS as a function of power prime and type of math calculation defined in an independently-defined ROI analysis, when controlling for individual differences in math confidence, math attitudes and anxiety.

**Figure 4:**
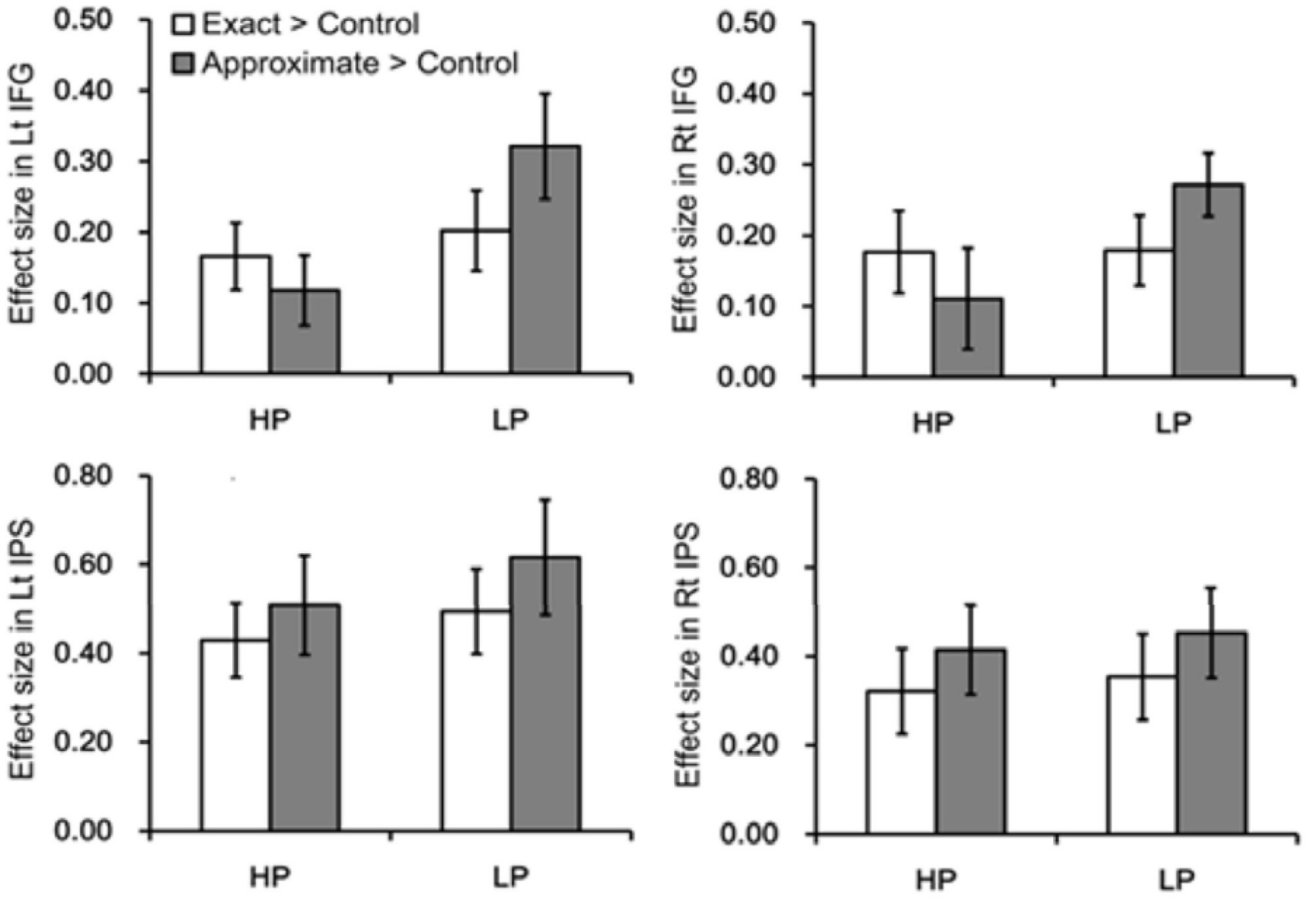
**Neural results from independent ROIs for exact and approximate math calculations as a function of high power (HP) and low power (LP) prime groups within both bilateral IFG and bilateral IPS**.

Within IFG, there was a significant power prime and type of math calculation interaction, *F*_(1, 18)_ = 9.21, *p* < 0.007, such that LP participants showed significantly greater neural response during approximate, but not exact, math calculation compared to HP participants, *t*_(22)_ = −2.30, *p* < 0.05. There was also a trend of a main effect of power prime, such that LP participants showed greater neural response compared to HP participants, *F*_(1, 18)_ = 3.45, *p* = 0.08 (Figure 4). Notably, within IPS, there was no interaction or main effect of power prime group on neural response during either exact or approximate math calculation (all *p*s > 0.05). There was no main effect of math confidence, math attitudes and anxiety on neural response within independently-defined IFG and IPS regions (Figure 4).

#### Regression analyses

To determine the extent to which social and personality factors predict neural response within bilateral IFG, we conducted a multiple linear regression with state-trait anxiety, math confidence, math attitudes and power prime as predictor variables. Results show that power prime β = 0.04, *t*_(18)_ = 2.28, *p* < 0.05, math confidence, β = 0.01, *t*_(18)_ = 2.38, *p* < 0.05, and trait anxiety, β = −0.12, *t*_(18)_ = −2.14, *p* < 0.05, but not state anxiety or math attitudes, uniquely predict neural response within bilateral IFG *R*^2^ = 0.62, *F*_(5, 23)_ = 5.78 *p* < 0.05.

## Discussion

Here we show for the first time that temporarily heightening a person's social power decreases neural response within regions previously associated with cognitive interference and improves math ability, particularly for approximate math calculation, even when controlling for individual differences in trait anxiety and math confidence. Specifically, people who are primed with LP are more likely to recruit left IFG when solving math problems, providing evidence that heightened cognitive interference during approximate math calculation may explain why math performance is decreased when people are in situations of LP. Furthermore, we speculate that power priming affects the neural processing during approximate compared to exact math calculation, due to incongruency with cognitive styles of math calculation. Our findings are consistent with prior behavioral studies demonstrating reduced executive functioning (Smith et al., 2008) and greater susceptibility to interference of extraneous information (Guinote, 2007) in LP relative to HP primed individuals when performing cognitive tasks. Hence, LP people may be more vulnerable to experiencing interference when trying to retrieve the approximate rather than exact answer, and thus require decreased recruitment of neural resources associated with cognitive interference in order to solve math problems accurately. Our findings demonstrate the importance of understanding how power priming affects math calculation not only at the behavioral, but also the neural level.

Notably, we also show that power priming increases females' recruitment of the left IFG during math calculation, irrespective of type of math calculation. Prior research has shown that when females are reminded of negative stereotypes about female's performance in math, they show increased recruitment of the ventral anterior cingulate cortex (vACC) during math calculation (Krendl et al., 2008), likely due to increased recruitment of social and emotional processing when reminded of their group's low status at math performance. By logical extension, an alternative possible interpretation of our findings is that females primed with LP may not only demonstrate greater cognitive inference when solving approximate compared to exact math calculation, but also show increased affective response which may also interfere with math calculation. For instance, in prior studies, we have previously shown that greater preference for egalitarianism increases empathic neural response with the left anterior insula, a subregion of the left IFG (Chiao et al., 2009b; Cheon et al., 2011). Furthermore, our current findings indicate that people with increased math confidence show greater neural response within left IFG. However, in the current study, we also show that neural response within the left IFG is negatively associated with individual differences in negative affect, specifically trait anxiety. That is, individuals who demonstrate greater trait anxiety actually show reduced recruitment of left IFG. Taken together, our findings indicate that increased recruitment of left IFG in LP compared to HP groups is not likely a result of increased affective response. Rather, power priming likely serves as a distinct kind of social influence on math calculation, reducing cognitive interference during math calculation, particularly when females are primed with high compared to LP.

Finally, we demonstrate that a novel power prime, specifically completing analogies that test knowledge of social power roles, in addition to writing a power prime essay, are effective at temporarily modulating both neural and behavioral responses during math calculation. Our findings have implications for interventions and procedures that may be implemented in educational studies and environments to improve math performance in social groups who are known to encounter negative cultural stereotypes about their groups' math ability. Recent evidence suggests that the human ability to perform numerical approximation is a foundational stepping stone for achieving more complex mathematical abilities. For instance, Halberda et al. (2008) recently demonstrated a robust correlation between non-verbal numerical approximation and math achievement, emphasizing the importance of honing this skill for future academic success. Here we show that the ability to experience math achievement may be modulated as a function of power priming. By temporarily heightening a person's sense of high or LP, we show that not only can math problems be solved with greater accuracy, but also that heightened cognitive interference, which is often thought of as one of the cognitive costs of stereotype threat during math calculation, can be reduced.

On a national scale, social status influences students' learning and future academic success. For instance, a substantially smaller proportion of high school seniors from low socioeconomic status (SES) households (50.8%) anticipate attaining post-secondary and graduate-level degrees in comparison to students from middle- and high-income households (66.4 and 86.6%, respectively) (US Department of Education, 2006). While this socioeconomic disparity in high school seniors' educational expectations may be due in part perceived or actual low SES, including a lack of access to resources, we propose that an additional facet of this dilemma is the absence of the psychological opportunity for under-privileged students to simply imagine themselves with high status situations or positions. Our findings suggest that classroom exercises that simply encourage students to imagine or act in positions of power or authority may prove effective in facilitating basic cognitive processes underlying multiple kinds of mathematical learning and help to close achievement gaps that exist between people from groups of varying social power.

### Conflict of interest statement

The authors declare that the research was conducted in the absence of any commercial or financial relationships that could be construed as a potential conflict of interest.

## Appendix

### Power analogies

**Team captain: Third string player**

1. Inexperienced
2. A leader
3. Studious
4. Silly

**Parent: Child**

1. Sleepy
2. Dependent
3. Ordinary
4. Powerful

**Prosecutor: Defendant**

1. Athletic
2. Compliant
3. Influential
4. Lighthearted

**Boss/Employer: Employee**

1. Clean
2. Compliant
3. Demanding
4. Hungry

**Resident advisor: Floor resident**

1. Fascinating
2. Materialistic
3. Authoritative
4. Submissive

**Senior: Freshman**

1. Gullible
2. Intimidating
3. Focused
4. Thorough

**Millionaire: Homeless person**

1. Destitute
2. Indecisive
3. Sincere
4. Pompous

**Interviewer: Job applicant**

1. Vulnerable
2. Compulsive
3. Have Leverage
4. Unpopular

**Judge: Lawyer**

1. Artistic
2. Esteemed
3. Submissive
4. Scientific

**Surgeon: Medical intern**

1. Subordinate
2. Adept
3. Lighthearted
4. Troubled

**Lawyer: Paralegal clerk**

1. Superior
2. Gentle
3. Subservient
4. Withdrawn

**General practitioner: Patient**

1. Authoritative
2. Helpless
3. Musical
4. Dull

**Frat/Sorority brother or sister: Pledge for frat/Sorority**

1. Submissive
2. Meditative
3. Patronizing
4. Environmentally Conscious

**Head of admissions committee: Aspiring incoming student**

1. Ashamed
2. Passive
3. Influential
4. Lonely

**Guard: Prisoner**

1. Powerless
2. Whimsical
3. Controlling
4. Precise

**Film director: Production assistant**

1. Forgetful
2. Important
3. Sensitive
4. Subordinate

**Pimp: Prostitute**

1. Controlling
2. A Daydreamer
3. Helpless
4. Sensitive

**Chief of surgery: Resident doctor**

1. Fashionable
2. Inferior
3. Superior
4. Theatrical

**CEO: Secretary**

1. Adventurous
2. Ethical
3. Prestigious
4. Subservient

**Editor-in-chief: Staff news writer**

1. Accomplished
2. Comical
3. Conservative
4. Subordinate

**Teacher: Student**

1. Knowledgeable
2. Fashionable
3. Impressionable
4. Clean

**Team coach: Team player**

1. Commanding
2. Cooperative
3. Impractical
4. Social

**Older sibling: Younger sibling**

1. Moral
2. Submissive
3. Disorganized
4. Dominant

**Chef: Dishwasher**

1. Accomplished
2. Unskilled
3. Forgetful
4. Frivolous
